# The ROX index as a predictor of standard oxygen therapy outcomes in thoracic trauma

**DOI:** 10.1186/s13049-021-00876-4

**Published:** 2021-06-21

**Authors:** Adrien Cornillon, Juliette Balbo, Julien Coffinet, Thierry Floch, Mathieu Bard, Guillaume Giordano-Orsini, Jean-Marc Malinovsky, Lukshe Kanagaratnam, Daphne Michelet, Vincent Legros

**Affiliations:** 1 Department of Anesthesiology and critical care, Reims University Hospital, Reims, France; 2 Surgical and Trauma Intensive Care Unit, Reims University Hospital, 45 rue Cognacq Jay, 51092 Reims Cedex, France; 3 University of Reims Champagne Ardennes, Reims, France; 4 Department of Emergency Medicine, Reims University Hospital, Reims, France; 5 Clinical Research Unit, Reims University Hospital, Reims, France

**Keywords:** Standard oxygen therapy, ROX index, Thoracic trauma, Outcome

## Abstract

**Background:**

Thoracic trauma is commonplace and accounts for 50–70% of the injuries found in severe trauma. Little information is available in the literature as to timing of endotracheal intubation. The main objective of this study was to assess the accuracy of the ROX index in predicting successful standard oxygen (SO) therapy outcomes, and in pre-empting intubation.

**Methods:**

Patient selection included all thoracic trauma patients treated with standard oxygen who were admitted to a Level I trauma center between January 1, 2013 and April 30, 2020. Successful standard SO outcomes were defined as non-requirement of invasive mechanical ventilation within the 7 first days after thoracic trauma.

**Results:**

One hundred seventy one patients were studied, 49 of whom required endotracheal intubation for acute respiratory distress (28.6%). A ROX index score ≤ 12.85 yielded an area under the ROC curve of 0.88 with a 95% CI [0.80–0.94], 81.63sensitivity, 95%CI [0.69–0.91] and 88.52 specificity, 95%CI [0.82–0.94] involving a Youden index of 0.70. Patients with a median ROX index greater than 12.85 within the initial 24 h were less likely to require mechanical ventilation within the initial 7 days of thoracic trauma.

**Conclusion:**

We have shown that a ROX index greater than 12.85 at 24 h was linked to successful standard oxygen therapy outcomes in critical thoracic trauma patients. It is our belief that an early low ROX index in the initial phase of trauma should heighten vigilance on the part of the attending intensivist, who has a duty to optimize management.

**Supplementary Information:**

The online version contains supplementary material available at 10.1186/s13049-021-00876-4.

## Background

Thoracic trauma (TT) is common place and accounts for 50–70% of the injuries found in severe trauma [1, 2]. The anatomical lesions arising from TT, such as pulmonary contusions, rib fractures, pneumothorax and hemothorax, are frequently manifold. TT is more commonly found in association with other traumatic injuries than in isolation [3].

Thoracic trauma goes hand in hand with significant morbidity and mortality in the intensive care unit (ICU).

Several scoring systems are useful for defining TT severity and include the injury severity score (ISS), the abbreviated injury scale (AIS) with particular focus on the thoracic region, and the thoracic trauma severity score (TTSS). These scores can also benefit clinicians as a means of predicting the risk of mechanical ventilation in TT patients [1, 4–6].

Various oxygenation modalities are available to intensive care unit (ICU) clinicians: conventional oxygen therapy via a nasal cannula, full-face and high concentration oxygen masks (SO), non-invasive ventilation (NIV), or high-flow nasal cannula (HFNC); and invasive mechanical ventilation (MV) [4–6].

Some studies have demonstrated the advantages of HFNC over conventional oxygen therapy in acute respiratory failure. HFNC enhances comfort, oxygenation and respiratory rates [7].

Selecting the best non-invasive oxygen therapy when managing TT remains a challenging task. In any event there should be no delay in administering invasive ventilation, otherwise an increase in ICU morbidity and mortality would prove inevitable [8]. Requirement of invasive MV must be foreseen by clinical criteria [2, 9].

There are no simple clinical criteria that can be used at the bedside to predict the respiratory evolution in TT patients. The ROX index, defined as: $$ \left(\begin{array}{c} SpO2\\ {} FiO2\\ {} RR\end{array}\right) $$, was elaborated by Roca et al. [10, 11]. The ROX index predicts HFNC failure in patients suffering from uniquely pneumonia-related respiratory distress.

The main objective of the present study was to assess the accuracy of the ROX index in predicting successful SO therapy outcomes and in pre-empting intubation in TT patients.

## Methods

### Study design

Admissions to the Surgical and Trauma ICU (Level 1 Trauma Center) at the University Hospital of Reims (France) were retrospectively analyzed from January 2013 to April 2020. This unit habitually accommodates all patients with suspected or confirmed serious trauma, occurring within a distance of 150 km in agreement with French trauma network policy [12].

**Fig. 1:**
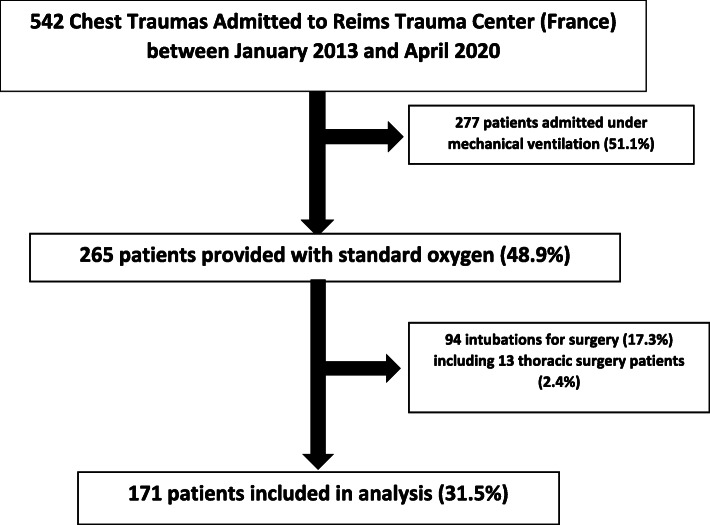
Flow Chart

All patients admitted to the ICU for TT (AIS thorax≥1) were screened regardless of oxygen requirements. Patients who received invasive MV on admission and those who were intubated for emergency surgery were excluded ***(***Fig. 1***)***. The ROX index was calculated on admission (H0), then at 2 (H2), 6 (H6), 12 (H12) and 24 (H24) hours in all included patients. Non-requirement of invasive MV was regarded as a successful SO therapy outcome.

### Data recording

Baseline demographic variables were collected including age, sex, trauma mechanism, isolated TT or polytrauma, severity scores (thorax AIS, ISS, SAPS II, TTSS), requirement for and duration of invasive MV, length of critical care unit stay and ICU mortality.

Mode of oxygen therapy delivery to patients (HFNC, NIV, SO) was collected in addition to oxygen saturation as measured by pulse oximetry and respiratory rate (RR).

Inspired fraction of oxygen (FiO2) was collected from ICU nursing charts that showed continuous hourly recording of HFNC and NIV. In the context of SO therapy, Fi02 was calculated in line with the Sequential Organ Failure Assessment formula [13] (20% + (4 x oxygen liter flow per minute), regardless of the interface used (nasal cannula, mask, or high concentration mask).

Intubation indication (surgery or acute respiratory failure) was also collected.

As were manifestation of acute respiratory distress syndrome (ARDS) consistent with the Berlin definition [13], nitric oxide administration, prone position [14] and patient requirement for veno-venous extracorporeal membrane oxygenation (VV-ECMO).

These data were used to calculate the ROX index (defined as the ratio of oxygen saturation as measured by pulse oximetry/FiO2 to respiratory rate) at H0, H2, H6, H12 and H24.

Multimodal analgesia was administered, including tier 1 analgesics and opioids (mainly via a patient-controlled morphine pump). Pain was assessed several times daily using the visual analog scale. Regional analgesia was delivered insofar as the patient was able to be moved and had no contraindications.

### Ethical considerations

This study was conducted in accordance with the Declaration of Helsinki and approved by the institutional review board (IRB) of the French Society of Anesthesiology and Critical Care (SFAR) (Approval number: IRB 00010254-- 2020-- 09).

STROBE (Strengthening the Reporting of Observational Studies in Epidemiology) guidelines were thoroughly followed [15]. (supplementary material).

### Statistical analysis

Quantitative variables were expressed as medians and interquartile range, categorical variables as frequency and percentages. Continuous variables were compared using the Student’s t-test or Mann-Whitney U test, as appropriate. Divergence across categorical variables was assessed via the chi-square or Fisher’s exact test, as appropriate. A receiver operating characteristic (ROC) curve was produced and optimal cut-off was defined by the Youden index.

Survival curves regarding higher and lower ROX index values were plotted using the Kaplan Meier method at 2, 4 and 7 days. The log-rank test was used for comparison of these curves.

Cox proportional hazards modeling was used to substantiate any association between the ROX index and requirement for invasive MV. Severity scores (SAPS II, ISS, AIS and TTSS) were additionally applied for adjustment.

Multivariate analysis by logistic regression was conducted to analyze the effect of ROX Index < 12.85 on SO therapy failure with adjustment for TTSS, simplified acute physiology score II (SAPS II) findings and chronic respiratory disease.

Inclusion of 170 patients was mandatory (to attain 50 SO failures) for sample size calculation based on multivariate analysis involving 4 variables.

Missing data was regarded as missing at random (MAR) and processed by mean imputation.

All analysis was performed using SAS, version 9.4 (SAS Institute, Inc., Cary, NC, USA) and R version 3.6.1 software.

## Results

### General characteristics of the included population

Between January 2013 and April 2020, 542 thoracic traumas were admitted to the Level 1 Trauma Center of the University Hospital of Reims (France). 277 patients (51.1%) were provided with invasive MV on admission. Of the remaining 265 patients (48.9%), 94 (17.3%) were placed on invasive MV for emergency surgery, which involved thoracic surgery in 13 (2.4%) patients (Fig. 1).

171 patients (31.5%) were included in the final analysis. Most of the patients were male (*n* = 134, 78.4%) with a median age of 48 years [31.1–67]. Road traffic accidents (87 patients [50.8%]) accounted for the most common trauma mechanism. Isolated TT was identified in only 38 cases (22.2%), and thoracic intercostal under water sealed drainage was required in 70 cases (40.9%). Median traumatic severity scores were 20 [13–25], 4 [3, 4], 8 [8–11] for ISS, AIS thorax and TTSS respectively.

49 patients (28.6%) required endotracheal intubation for acute respiratory distress within a mean time interval of 33 h (−/+ 53) following ICU admission. ICU length of stay was 4 [2–9] days, and 9 patients died on the ICU (5.3%) **(**Table 1**)**.

**Table 1:** Baseline Characteristics

	*N* = 171
Age (years)	48 [31.1–67]
Sex, male, n (%)	134 (78.4)
Chronic respiratory disease, n (%)	12 (7)
Mechanism of Trauma, n (%)	
Road traffic accident	87 (50.8)
Hit by object	16 (9.3)
Fall/jump	62 (36.2)
Assault	6 (3.5)
Penetrating trauma, n (%)	6 (3.5)
Isolated chest trauma, n (%)	38 (22.2)
Thoracic intercostal under water sealed drain n (%)	70 (40.9)
Injury severity score (ISS)	20 [13–25]
Abbreviated injury scale (*AIS)* thorax	4 [3–4]
Thoracic trauma severity score (TTSS)	8 [5–11]
Simplified acute physiology score II (SAPS II)	21 [13–33]
Acute respiratory failure, n (%)	49 (28.6)
Time to intubation (hours)	33 ± 53
Acute respiratory distress syndrome (ARDS), n (%)	24 (14)
Prone positioning, n (%)	8 (4.7)
Nitric oxide, n (%)	1 (0.6)
Extracorporeal membrane oxygenation, n (%)	2 (1.2)
Length of ICU stay (days)	4 [2–9]
ICU deaths, n (%)	9 (5.3)

Data are expressed as medians [interquartile], counts (percentage) or means ± standard derivation

### Variables associated with SO failure and ROX index

The ROX index was significantly lower at different collection times (H0, H2, H6, H12, H24) in the failed SO therapy group in which a significantly divergent median ROX per 24 h of 8.4 [4.9–11.7] vs. 17.8 [14.4–22.1], *p* <  0.0001 was observed.

A link was established between age and SO therapy failure (47 years [28.6–63.8] vs. 56.7 years [41.2–72], *p* = 0.02). Significant correlation was also found between SO therapy failure and severity scores regarding both trauma and overall severity scores. Treatment by thoracic drainage tube was more commonly required in the failure group (*n* = 34, 69.4%) vs. successful outcome group (*n* = 36, 29.5%), *p* <  0.0001.

ICU mortality was higher in the group that received MV: 8 (16.3%) vs. 1 (0.82%), p <  0.0001 **(**Table 2**).**

**Table 2:** Standard Oxygen Outcomes

	Success	Failure	***p value***
		(***n*** = 122)	(***n*** = 49)
Age (years)	47 [28.6–63.8]	56.7 [41.2–72]	**0.02**
Sex, male, n (%)	95 (77.8)	39 (79.6)	0.8
Chronic respiratory disease, n (%)	6 (4.9)	6 (12.2)	0.08
Isolated chest trauma, n (%)	26 (21.3)	12 (24.5)	0.6
Thoracic intercostal under water sealed drain n (%)	36 (29.5)	34 (69.4)	**< 0.0001**
Severity score:			
- ISS	17 [13–24]	20 [16–29]	**0.0001**
- *AIS* Thorax	3 [3–4]	4 [4–4]	**0.003**
- TTSS	8 [4–10]	12 [9–14]	**< 0.0001**
- SAPS II	17 [11–25]	34 [16–29]	**< 0.0001**
HFNC required in initial 24 h, n (%)	0 (0)	6 (12.2)	**< 0.0001**
NIV required in initial 24 h, n (%)	2 (1.6)	0 (0)	**< 0.0001**
Rox Index			
H0	15.5 [11.7–20.8]	7.3 [4.9–10.6]	**< 0.0001**
H2	17.6 [12.9–21.4]	10.7 [5.3–14.8]	**< 0.0001**
H6	17.8 [14.6–23.1]	11.1 [7.9–18.5]	**0.001**
H12	18.2 [15.1–22.8]	2.9 [7.8–18.5]	**0.0013**
H24	18.2 [14.9–23.8]	13.6 [7.9–16.6]	**0.0013**
Mean/24 h	17.8 [14.4–22.1]	8.4 [4.9–11.7]	**< 0.0001**
ICU deaths, n (%)	1 (0.82)	8 (16.3)	**< 0.0001**

Data are expressed as medians [interquartile] or counts (percentage), *HFNC* High flow nasal cannula, *NIV* Non-invasive ventilation

### ROC curve

A ROC curve was plotted before selecting ROX index cut-offs that made a more accurate prediction of SO therapy failure. The area under the ROC curve was 0.88 with a 95% CI [0.80–0.94].

**Fig. 2:**
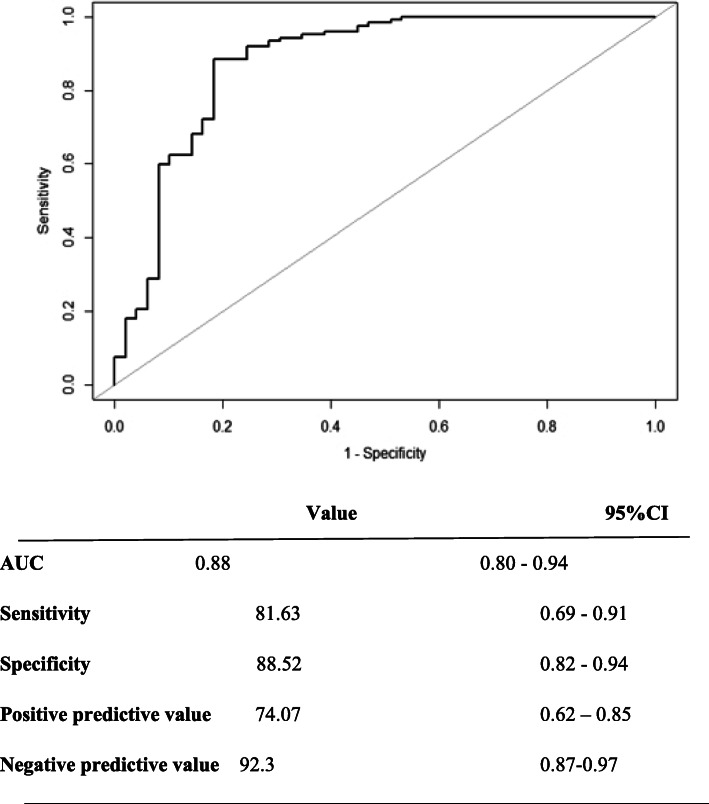
ROC curve for Rox index cut-off of at 12.85. *ROC = receiver operating characteristic; AUC = area under curve; CI = confidence intervals*

A ROX index cut-off value of 12.85 yields sensitivity at 81.63, 95%CI [0.69–0.91], specificity at 88.52, 95%CI [0.82–0.94], a positive prospective value of 74.07% [0.62–0.85], a negative predictive value of 92.3% [0.87–0.97] and a Youden index of 0.70 (Fig. 2).

### Survival analysis

**Fig. 3:**
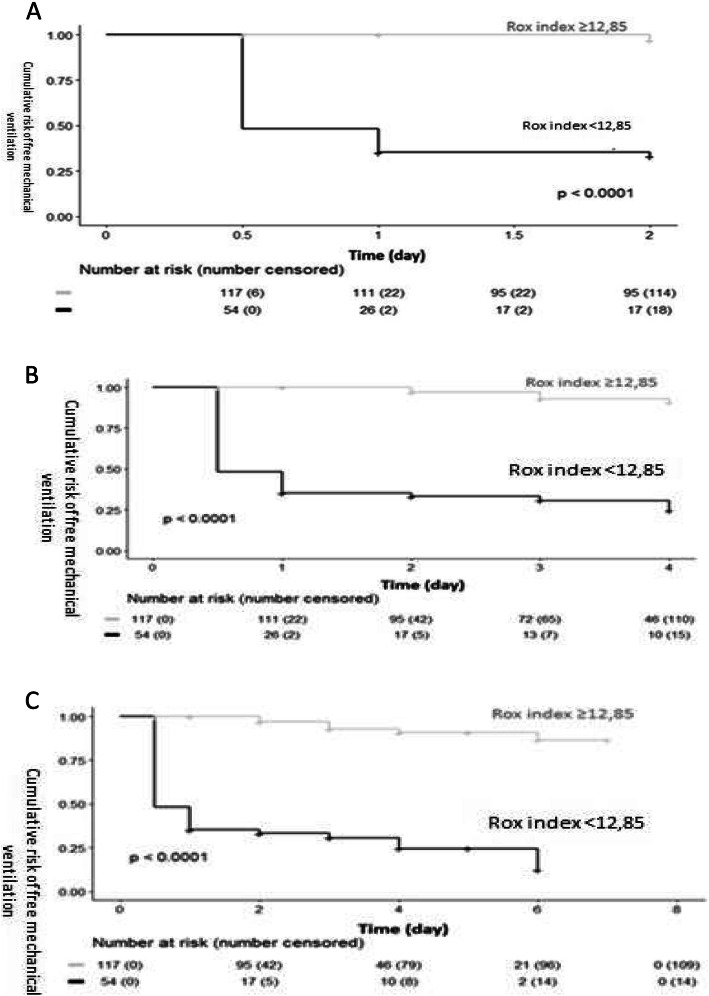
Kaplan-Meier Curves. Kaplan-Meier curves at Day 2 (A), day (B), and Day 7 (C)

Kaplan-Meier curves at days 2, 4 and 7 consistent with a ROX index cut-off value of 12.85 are shown in Fig. 3.

MV was significantly more frequent in patients with a ROX index of less than 12.85 (*p* <  0.0001) (Fig. 3).

A Cox proportional hazards model was applied to substantiate a relationship between the ROX index of thoracic trauma patients on SO therapy and the risk of MV requirement.

Patients with a mean ROX index greater than 12.85 within the initial 24 h had less requirement for MV in the first 7 days post thoracic trauma, even after adjustment for various trauma severity scores (ISS, AIS thorax, TTSS) including adjustment for overall severity scores (SAPS II) **(**Table 3**).**

**Table 3:** Cox proportional hazards model (Cox Regression) for analysis of effect of ROX Index ≥12.85 on SO at days 2, 4 and 7 and severity scores

	HR	95%CI	*P*-value
Unadjusted ROX index ≥12.85			
At day 2	0.03	0.009–0.095	< 0.0001
At day 4	0.06	0.025–0.129	< 0.0001
At day 7	0.06	0.027–0.129	< 0.0001
Adjusted by ISS			
At day 2	0.03	0.010–0.106	< 0.0001
At day 4	0.06	0.034–0.141	< 0.0001
At day 7	0.06	0.029–0.137	< 0.0001
Adjusted by AIS thorax			
At day 2	0.03	0.010–0.106	< 0.0001
At day 4	0.06	0.027–0.139	< 0.0001
At day 7	0.06	0.029–0.136	< 0.0001
Adjusted by TTSS			
At day 2	0.04	0.012–0.142	< 0.0001
At day 4	0.07	0.034–0.187	< 0.0001
At day 7	0.08	0.036–0.182	< 0.0001
Adjusted by SAPS II			
At day 2	0.04	0.013–0.146	< 0.0001
At day 4	0.08	0.034–0.190	< 0.0001
At day 7	0.06	0.029–0.136	< 0.0001

*ISS* Injury severity score; *AIS* Abbreviated injury scale; *TTSS* Thoracic trauma severity score; *SAPS II* Simplified acute physiology score II

### Multivariate analysis

Multivariate analysis performed on ROX index < 12.85 adjusted for age, TTSS, SAPS II, and IRC found an OR of 9.86 95% CI (3.31–29.40) *p* <  0.001 **(**Table 4**).**

**Table 4:** Multivariate analysis to substantiate relationship between ROX Index < 12.85 and SO failure

	***OR***	95%CI	***p-value***
**ROX index < 12.85**	9.869	3.313–29.402	<.0001

Odds ratio adjusted for age, thoracic trauma severity score (*TTSS*), simplified acute physiology score II (*SAPS II*) and chronic respiratory disease

## Discussion

This study reports the first diagnostic performances of the ROX index in predicting successful SO therapy outcomes in TT patients on the ICU. The research work focused on a Level I Trauma Center, reinforcing our cohort which in all probability reflects the most severe cases of thoracic trauma in whom using the ROX index makes sense. A ROX index ≥12.85 at 24 h was predictive of a successful SO therapy outcome. This threshold remained significant even after adjustment involving the various severity scores (AIS thorax, ISS, TTSS, and SAPS II) at day 2, day 4 and day 7.

This threshold is much higher than the one initially suggested by Roca et al. (≥ 4.88 at hour 12). A possible explanation for such discrepancy is the type of patients involved, which was significantly different in our retrospective cohort. The present study was based on the assessment of a large number of patients who had sustained thoracic trauma. Most of these patients were young, male and had no respiratory comorbidity. In stark contrast to Roca et al. (35% of the patients suffered from chronic respiratory insufficiency) [11].

Our populations were likely better able to tolerate their thoracic injuries on account of their healthy pulmonary parenchyma. Additionally, analgesia - one of the priorities when managing TT - could account for a higher ROX index. Few patients received locoregional analgesia partly due to severe polytrauma-induced coagulopathy [16, 17]. This may have resulted in morphine consumption, induced respiratory-depressing effects and caused a decrease in respiratory rate, leading to an increased ROX threshold in TT patients. With a view to ruling out this bias, it would be worthwhile to conduct a study that has a similar analgesia protocol but includes regional anesthesia to obtain data on opioid dosage.

A higher ROX index could suggest TT patients require faster invasive MV than patients suffering from pneumonia alone. One of the reasons for this may reside in the pain and accompanying damage endured, all of which are relevant factors for clinicians involved in intubation decision-making.

In common with Roca, we produced ROX cut-off points for TT with high specificity, facilitating early identification of patients prone to risk of standard oxygenation failure.

Optimal timing of intubation is a constant cause of concern for clinicians. Late intubation has been linked to poorer outcomes in patients with acute respiratory failure [18]. Invasive MV in TT patients is related to longer ICU stays, pneumonia, infection rates and mortality [19].

Data surrounding invasive MV highlight the complexity of discerning whether or not to intubate a TT patient, especially since strong predictive factors to underpin decision-making are nonexistent. A ROX index ≥12.85 at 24 h applied to TT cases could be a valuable tool in tackling this challenge.

The ROX index has also been addressed in relation to various management time intervals (H0, H2, H6, H12, and H24). A significant threshold was observed for each of these intervals. These results suggest that the ROX index could be used at an earlier stage in TT management. It would also be worthwhile to study the applicability of this index in pre-hospital care settings following analgesia, particularly in regions where a nearby trauma center is lacking. Larger prospective studies will be required to confirm these results.

French guidelines recommend using non-invasive ventilation (NIV) in TT and hypoxemic patients [3].

There is a dearth of data assessing HFNC in this indication. NIV likely provides better protection against atelectasis formation that is a common complication of thoracic injury [20]. However, these data require confirmation in a bigger cohort on account of the small patient numbers receiving this type of treatment in our study. Antonelli et al. showed that PaO2/FiO2 < 146 within 1 h of NIV was an independent factor for requiring intubation. This has been demonstrated in patients suffering from acute respiratory distress, 25% of whom had sustained TT [8]. The fact that so few patients received NIV at 24 h in this retrospective cohort is unfortunate and restricts the scope of the ROX index.

It is conceivable that a different threshold would come to light in patients treated with NIV. It is our belief that a prospective observational study placing emphasis on the ROX index and NIV would be worthwhile.

In the present study, median time to intubation was 33 h +/− 53 and likely stems from the complexity and different mechanisms of TT (parietal lesions, vessel damage, organ injury). This time interval indicates that the condition of TT patients is not always critical straightaway but can deteriorate further down the line. It is highly likely that bronchial obstruction, atelectasis formation and an increase in contusions, weak cough and respiratory exhaustion accounted for such deterioration. It is noteworthy that mortality was significantly higher in the SO failure group. Several variables such as the clinical severity of the initial injury report (AIS Thorax, ISS, TTSS, SAPS II) could provide an explanation for these findings, but overly late intubation may have aggravated underlying lesions and favored this dimension of poor outcome.

In addition, although patients intubated for neurological coma were excluded, no data was collected regarding brain injuries that potentially aggravate respiratory function.

The three major features of TT typically reported are: contusions, pulmonary wounds and parietal lesions. These different features can each lead to respiratory distress even if the pathophysiology involved varies. Pulmonary contusions result from alveolar-capillary rupture which may develop into pneumonia over several days, entailing significant mortality in intensive care [21, 22]. Lung wounds mainly caused by ballistic or stab wounds present as both open and closed thoracic wounds. They often involve lesions to the large vessels or the intrathoracic organs (heart, aorta, pericardium, pleura) with initial hemorrhagic shock [23, 24]. These patients require urgent surgery, rendering futile any application of the ROX index.

While thoracic wall lesions lead to problems with the mechanics of ventilation, flail chest involving paradoxical breathing is the most severe parietal lesion. It does not strictly speaking lead to respiratory distress but can develop into respiratory exhaustion [25, 26].

Few patients were treated surgically for stabilization of costal lesions but were excluded from the study either because they were scheduled for intubation or had already been intubated on admission. Recent studies have explored reducing the duration of MV and length of resuscitation stay in flail chest patients [27, 28]. A French multicenter study is currently underway with the aim of substantiating these encouraging results. Similarly, the respective benefits of early surgical management (within 48 h) or later surgical management (5–10 days) are not clearly defined [29–32]. The ROX index could be advanced as a guide for surgical management of isolated TT.

Attention should however be drawn to certain limitations. Firstly, our monocentric retrospective study was conducted over 8 years, during which time there were advances in trauma treatment practice, thus modifying the criteria for MV. Larger multicenter validation studies are needed to establish the role of the ROX index in TT.

Secondly, time of intubation is left to the discretion of the treating clinician in the absence of any formally written protocol based on clinical and biological criteria. A post-hoc analysis was carried out on the basis of patient health record analysis confirming the indication for intubation; the expert estimated that 45 of the 49 intubated patients actually required this procedure.

Thirdly, we are aware that calculation of FiO2 under SO therapy lacks accuracy. As a patient’s inspiratory flow rate is higher than the gas flow rate administered by the device, the oxygen administered via the device is mixed with ambient air (FIO2 at 21%) and the resulting FIO2 cannot be measured. Higher patient rates of tachypnea with high inspiratory flow result in greater variation in the ratio of ambient air to enriched air. In the light of these physical phenomena, FiO2 percentages in SO are overestimated. This translates into a similar link between respiratory rate and a lower ROX index which is necessarily a cause of concern for intensivists.

Finally, management of oxygen therapy and mode of administration were governed not by a titration protocol but by a minimum oxygen saturation target based on patient characteristics.

## Conclusion

We have shown that a ROX index greater than 12.85 at 24 h was linked to successful standard oxygen therapy outcomes in critical thoracic trauma patients.

In our opinion, early low ROX index in the initial phase of trauma should heighten vigilance on the part of the attending intensivist, who has a duty to optimize management (thoracic tube insertion, NIV and particular focus on regional anesthesia).

## Supplementary Information

**Additional file 1.**

## Data Availability

The datasets used and/or analysed during the current study are available from the corresponding author on reasonable request.
